# Riemann solvers and Alfven waves in black hole magnetospheres

**DOI:** 10.1186/s40668-016-0018-1

**Published:** 2016-09-13

**Authors:** Brian Punsly, Dinshaw Balsara, Jinho Kim, Sudip Garain

**Affiliations:** 1 1415 Granvia Altamira, Palos Verdes Estates, CA 90274 USA; 2 ICRANet, Piazza della Repubblica 10, Pescara, 65100 Italy; 3 Physics Department, University of Notre Dame du Lac, 225 Nieuwland Science Hall, Notre Dame, IN 46556 USA

**Keywords:** black hole physics, magnetohydrodynamics (MHD), galaxies: jets, galaxies: active, accretion, accretion disks

## Abstract

In the magnetosphere of a rotating black hole, an inner Alfven critical surface (IACS) must be crossed by inflowing plasma. Inside the IACS, Alfven waves are inward directed toward the black hole. The majority of the proper volume of the active region of spacetime (the ergosphere) is inside of the IACS. The charge and the totally transverse momentum flux (the momentum flux transverse to both the wave normal and the unperturbed magnetic field) are both determined exclusively by the Alfven polarization. Thus, it is important for numerical simulations of black hole magnetospheres to minimize the dissipation of Alfven waves. Elements of the dissipated wave emerge in adjacent cells regardless of the IACS, there is no mechanism to prevent Alfvenic information from crossing outward. Thus, numerical dissipation can affect how simulated magnetospheres attain the substantial Goldreich-Julian charge density associated with the rotating magnetic field. In order to help minimize dissipation of Alfven waves in relativistic numerical simulations we have formulated a one-dimensional Riemann solver, called HLLI, which incorporates the Alfven discontinuity and the contact discontinuity. We have also formulated a multidimensional Riemann solver, called MuSIC, that enables low dissipation propagation of Alfven waves in multiple dimensions. The importance of higher order schemes in lowering the numerical dissipation of Alfven waves is also catalogued.

## Introduction

In this century, there has been great progress in 3-D magnetohydrodynamic (MHD) simulations of black hole magnetospheres (De Villiers et al. 2003; Komissarov 2004; Komissarov 2005; Hawley and Krolik 2006; Fragile et al. 2007; McKinney and Blandford 2009; McKinney et al. 2012). To varying degrees, each of these simulations require knowledge of the 1-D characteristics of the MHD system in order to time evolve the magnetosphere. Specifically, the polarization properties of the waves determine the changes in the fields that can be propagated at the appropriate speed along a particular characteristic direction. In single fluid ideal MHD there are three plasma modes in the system, the fast mode, the Alfven (or intermediate) mode and the slow mode. Black hole magnetospheres have the property that all plasma must pass progressively through the slow, Alfven and fast critical surfaces before reaching the event horizon (Punsly 2008). As each critical surface is crossed, the unique information associated with each wave mode is unable to be communicated upstream to an outgoing wind or jet. The event horizon wind system has no boundary conditions at its terminus, there are asymptotic infinities both at the event horizon and at large radial coordinate (Punsly 2008). There are only lateral boundary conditions imposed by accreting gas. Thus, the wind system itself and the lateral boundary conditions determine 3-D single fluid perfect MHD wind solutions. Furthermore, due to the paired wind nature of the event horizon wind system (an ingoing accretion inner wind and the outgoing outer wind or jet), plasma is always drained off of the field lines and auxiliary physics (mass floors) must be injected by hand in order to keep numerical simulations from crashing at low density. Mass floors are a source of MHD waves and are generally chosen to enhance dissipation. Consequently, there are many unique aspects to the application of MHD that can influence the final steady state of the wind system. Describing the evolution of the event horizon magnetosphere with single fluid MHD is wrought with non trivial subtleties.

These subtleties relate to the numerical determination of the field line angular velocity as viewed from asymptotic infinity, $\varOmega _{F}$. This is of primary interest since the Poynting flux of the wind scales as $\varOmega _{F}^{2}$ (Punsly 2008). First, contrary to previous claims of early simulations, newer simulations indicate that $\varOmega _{F}$ can be altered significantly by the auxiliary method of injecting plasma (McKinney et al. 2012; Beskin and Zheltoukhov 2103). We consider the unique role of the oblique Alfven wave in this process. A unique component of the momentum flux is propagated along the Alfven characteristics and this momentum flux is a component of the MHD equations written in conservative form. It is also the only isolated discontinuity that propagates a physical charge. Black hole magnetospheres that support an outgoing relativistic jet, rotate and have a Goldreich-Julian charge density. The Alfven critical surface for the inflow (IACS) is quite far from the event horizon. For rapidly rotating black holes and the most recent $\varOmega _{F}$ values from numerical simulations, the proper distance is 2 to 3 black hole radii from the event horizon. For proper evolution of the magnetic field rotation rate and the induced charge density, one must be able to simulate the role of Alfven waves with high fidelity both globally and inside of the IACS. Thusly motivated, we discuss in this article new numerical methods that are designed to accurately characterize the Alfven wave numerically in the rarefied environment of black hole magnetosphere.

An accurate depiction of the time evolution of a black hole magnetosphere and the global considerations germane to the IACS are intimately related to minimizing the numerical dissipation of Alfven waves. The IACS is a one-way surface as far as t he propagation of Alfven waves is concerned. In other words, at the IACS and within it, all Alfven waves should propagate inwards and only inwards. The propagation of waves in a higher order Godunov code is modulated by the Riemann solver. It is, therefore desirable if the Riemann solver can mimic this one-way propagation property for Alfven waves. Alas, whether a Riemann solver does so or not, depends crucially on the design of the Riemann solver. Some Riemann solvers which retain the substructure associated with Alfven waves within the Riemann fan, can indeed represent such a one-way propagation of Alfven waves. Other Riemann solvers, like the HLL Riemann solver, do not retain the substructure associated with the Alfven waves. At extraordinarily high resolutions, any well-designed code will of course minimize this dissipation. However, the present generation of simulations have all been done with low or modest resolutions. Furthermore, they have mostly used the HLL Riemann solver which, we argue, applies a maximal, and deleterious, dissipation to Alfven waves. To appreciate this point, realize that the HLL Riemann solver is based on a wave model that has just two extremal waves. These two extremal waves determine the ends of the Riemann fan in one-dimension. The speed of these extremal waves is usually set to the extremal signal speeds in the physical problem. For a relativistic MHD (RMHD) simulation of highly magnetized event horizon magnetospheres, these extremal signal speeds are usually set to approximately the speed of light propagating in either direction at a zone interface where the Riemann solver is applied. The HLL Riemann solver does not incorporate any further sub-structure associated with intermediate waves. Consequently, the HLL Riemann solver maximizes the dissipation of Alfven waves even near the IACS. This is the very location where the dissipation of these waves has to be minimized. Introducing the intermediate waves in the Riemann fan reduces the dissipation, but that effect is not incorporated in the HLL Riemann solver.

There is another issue that increases the dissipation of Alfven waves, in more than one dimension. Riemann solvers applied to black hole magnetospheres have been treated as 1-D in each direction. However, a true multi-dimensional Riemann solver has a strongly interacting region in which the numerical fluxes in orthogonal directions become intertwined (Balsara 2012). Careful treatment of the strongly-interacting region results in far less dissipation (as we show for RMHD in Section 4). The increase in computational complexity associated with a multidimensional Riemann solver is handily offset by larger timesteps and greater code robustness. The accuracy of the numerical depiction of the role of the Alfven waves near a black hole during jet production is facilitated by a true multidimensional scheme that incorporates the strongly interacting region.

Our ultimate goal is to understand the detailed time evolution of black hole magnetospheres. This is subtle because one must try to understand in each time step how the mass floor is affecting the time evolution. Thus, the transient structure is essential to monitor in order to see how transients associated with plasma injection are altering the time evolution of the system. This requires an inherently very stable numerical scheme (‘well-balanced’, which we describe below). Furthermore, we note that the simulations of Komissarov (2004) and Komissarov (2005) did tend to a steady state and this made it possible to carry out convergence testing for those simulations. However, several simulations seemed to never reach a steady state and, therefore, one cannot carry out convergence studies for them. In particular, an unexpected finding of Krolik et al. (2005) was that the event horizon magnetospheres in numerical simulations are very unsteady and appear to be more like a cauldron of strong MHD waves rather than a force-free structure. ‘For example, although the funnel region is magnetically-dominated, it is not in general in a state of force-free equilibrium. Indeed, the very large fluctuations that continually occur in the outflow show that it is never in any state of equilibrium, force-free or otherwise.’ This also appears to be the case in the simulations of McKinney et al. (2012) based on the supporting online movies. This suggests that wind formation in event horizon magnetospheres might be subject to large numerically induced transients which would mask the kinds of effects that we are looking for. Alternatively, if these large transients are integral to jet formation it indicates a dynamic in which strong waves from the lateral boundaries created by the accretion flow scatter off the event horizon magnetosphere producing strong gusts of energy in a jetted outflow.

It is important to separate these potential physical effects from numerical effects. However, schemes that are based on higher order Godunov methods, especially those that are based on the HLL Riemann solver, are notorious for not achieving steady state even when the physical problem admits such a steady state! This was first observed when higher order Godunov schemes with Riemann solvers were first applied to metreological simulations (which simulate wind flow in the earth’s atmosphere) or to the shallow water equations (which simulate lake and ocean circulation) (Parés 2006). Unless the numerical scheme has a special property called well-balancing, it usually does not find a stationary state even when one exists. Instead, the simulation generates ‘numerical storms’ - high velocity flows that are entirely a numerical artefact. The issue of well-balancing has recently become more compelling within the context of astrophysical flows with the work of Kappeli and Mishra (2014, 2016). Within the context of Type IIb supernova simulations, it has been found that the proto-neutron stars refuse to reach steady state if the scheme is not well-balanced, i.e., the numerical method has to be specially engineered to that it can find a steady-state proto-neutron star solution if one exists. Kappeli and Mishra have also explored time-dependent simulations that are not in steady state. Their very interesting result is that even for simulations that have no reason to be in steady state, the inclusion of well-balancing provides a substantial improvement in the accuracy of the simulation. This result has bearing on the black hole magnetospheres problem because it suggests that even when the simulations are far from steady state they might indeed be helped by well-balancing.

In order to verify that one has a well-balanced scheme, one has to know what the steady states of the system are and make sure that the simulated system is driven towards that steady state. This does not mean that every system must reach a steady state; it only means that when such a steady state exists, the numerical code is equipped to find it. For a scheme to be truly well-balanced, it needs to have two attributes. First, the reconstruction procedure has to be modified so that any potential steady state solution is subtracted off from the reconstructed solution. Second, the scheme must use a Riemann solver that can capture intermediate waves - specifically the contact discontinuity in classical Euler flow. We admit, that in certain circumstances it may not be possible to identify the steady state solution that has to be subtracted off. In such situations, the numerical scheme should at least be well-balanced up to second order. In practical terms, this means that a modest resolution simulation will not find the steady state solution to machine accuracy. However, it will nevertheless find the steady state solution with accuracies that are proportional to the size of the mesh squared. Being well-balanced up to second order is a weaker notion of well-balancing compared to being truly well-balanced. In order for a scheme to be well-balanced up to second order, it is imperative that the Riemann solver should at least capture stationary contact discontinuities in a self-gravitating situation involving Euler flow. By extension, any MHD code that is capable of capturing stationary equilibria up to second order should at least be based on a Riemann solver that captures contact discontinuities and Alfven waves. The discussion in this paragraph has made it clear that in order to disentangle a flow that is chaotic because of the physics of the situation from a flow that is chaotic because of simple numerical effects, one must pay attention to the form of the Riemann solver.

In summary, for the purposes of exploring the time evolution of event horizon magnetospheres, we require a well-balanced scheme to second order that will eliminate recurrent large transients. This is facilitated by preserving the Alfven wave as discussed above, but also requires that the inclusion of the contact discontinuity in the Riemann fan. Furthermore, without the contact discontinuity, the huge density gradient between accretion flow and the evacuated funnel of the event horizon magnetosphere means that plasma flows into the funnel by numerical diffusion. This is far from ideal if we want to explore the role of mass injection on the final solutions. To improve this situation, one desires a Riemann solver that at least minimizes the dissipation at contact discontinuities. (For more helpful information on numerical schemes and Riemann solvers of relevance to astrophysics, please visit http://www.nd.edu/~dbalsara/Numerical-PDE-Course.)

Designing low-dissipation Riemann solvers for RMHD is a challenging enterprise. An exact RMHD Riemann solver exists (Giacomazzo and Rezzolla 2006; Giacomazzo and Rezzolla 2007), but it is not practicable for use in numerical codes. HLLC Riemann solvers for RMHD exist (Mignone and Bodo 2006; Honkkila and Janhunen 2007; Kim and Balsara 2014). They enable a stationary contact discontinuity to be captured on a mesh. However, they dissipate stationary Alfven waves just like an HLL Riemann solver. HLLD Riemann solvers for RMHD (Mignone et al. 2009), do exist, which enable stationary contact discontinuities as well as stationary Alfvenic discontinuities to be captured on a computational mesh. Unfortunately, the method is iterative, which makes it computationally very expensive. Furthermore, when an iteration fails to converge, the method becomes brittle. With the emergence of the HLLI Riemann solver (Dumbser and Balsara 2016), it has become possible to capture stationary contact discontinuities as well as stationary Alfvenic discontinuities using a Riemann solver that is non-iterative and computationally inexpensive. The first goal of this paper is to document this capability in the astrophysical literature.

RMHD simulations also have to maintain the divergence-free structure of the magnetic field. This necessitates the use of a Yee-type mesh where the magnetic field components are specified at the faces of the mesh and the electric fields are to be evaluated at the edges of the mesh. It was claimed by Gardiner and Stone (2005), Gardiner and Stone (2008), Beckwith and Stone (2011), White and Stone (2015) that stabilizing the evolution of the magnetic field requires that one should always double the dissipation in the electric field at every timestep. Unfortunately, that approach has been used in the RMHD literature with the result that the already excessive dissipation of the HLL Riemann solver is increased even further in simulations. Such explorations ignore recent advances in multidimensional Riemann solver technology (Balsara 2010; Balsara 2012; Balsara 2014; Balsara 2015; Balsara et al. 2014; Balsara and Dumbser 2015; Balsara et al. 2016a). In a recent paper, Balsara and Kim (2016), showed that an exact analogue of the HLLI Riemann solver in multidimensions can be designed for RMHD. Their work is based on the original paper by Balsara et al. (2016b). Such multidimensional Riemann solvers go under the name of MuSIC Riemann solvers. Here the MuSIC acronym stands for Multidimensional, Self-similar strongly-Interacting Riemann solver that is based on Consistency with the conservation law. By introducing substructure associated with the multidimensional propagation of Alfven waves, the MuSIC Riemann solver reduces the dissipation of Alfven waves that propagate at any angle with respect to the mesh. The second goal of this paper is to catalogue the advantages of the MuSIC Riemann solver in reducing the dissipation involved in the multidimensional propagation of Alfven waves in RMHD.

In this article, we claim that modern 5-wave Riemann solvers can now be implemented that can allow a systematic assessment of the issues related to determination of $\varOmega _{F}$. This would require the formulation and simulation of simple magnetospheres, lateral boundary conditions and plasma injection mechanisms. Proper time evolution of the 3-D magnetosphere requires two main aspects of the solver, low dissipation and well balancing. We have motivated both these issues in the previous paragraphs. We demonstrate that the new Riemann solvers described here are capable of delivering on these goals in the following.

Riemann solvers offer one way of reducing numerical dissipation and Riemann solvers that preserve essential features of the flow are certainly central to many aspects of jet simulation. Recent advances in higher order schemes has made it possible to go beyond the traditional second order Godunov scheme that is commonplace in computational astrophysics. The third goal of this paper is to show that higher order schemes for RMHD do exist (Balsara and Kim 2016; Del Zanna et al. 2007; Zanotti and Dumbser 2016), which make it possible to go beyond second order of accuracy. We show that the combination of higher order schemes and appropriate Riemann solvers can go a long way towards enabling almost dissipation-free propagation of Alfven waves.

The paper is organized as follows. In Section 2, we discuss dissipation inside the IACS in Riemann solvers in general terms. It is shown that the HLLI Riemann solver provides the theoretical minimum dissipation that is consistent with a stable numerical scheme. In Section 3, we demonstrate by explicit examples that the HLLI RMHD Riemann solver preserves the Alfven wave with high accuracy and respects the contact discontinuity. In Section 4, we incorporate the important aspects of multi-dimensionality with the MuSIC RMHD Riemann solver. This is truly a multi-dimensional scheme and we demonstrate that its ability to resolve the strongly interacting region substantially reduces Alfvenic dissipation compared to higher dimensional schemes that utilize 1-D Riemann solvers in each direction. In our final discussion section, we describe how a numerical scheme that utilizes the MuSIC Riemann solver would be suitable for specialized simulations that would shed light on the causal physics of the time evolution of event horizon magnetospheres and help define the full panoply of physically allowed and disallowed solutions.

## Dissipation in Riemann solvers

In this section, we illustrate the mathematical implications of the IACS in conservative upwind schemes that utilize Riemann solvers. (Please also note that schemes that do not use Riemann solvers necessarily have to introduce even higher levels of dissipation. This is because they cannot discriminate between wave families in the way that some of the better Riemann solvers can.) For simplicity and without loss of generality, consider a one-dimensional grid. The conservation law that must be solved in each direction and at each time step can be formally written as

1 $$ \frac{\partial U}{\partial x^{0''}} + \frac{\partial F}{\partial x^{1''}} =0 . $$

For a fine enough mesh and a well behaved coordinate system, the covariant derivatives can be replaced with ordinary derivatives in the conservation equation. In fact, RMHD Riemann solvers are used in GRMHD (general relativistic MHD) simulations (Komissarov 2004; Gammie et al. 2003; Etienne et al. 2015). In an integral (weak solution) solution of the Riemann problem, the higher order corrections due to connection coefficients will be small (bilinear) corrections compared to the integral of the derivative terms which are linear in the space-time mesh size. This is the essence of the validity of ignoring the source (connection coefficient) terms in the GRMHD Riemann solvers and is a manifestation of the equivalence principle. We note that in the GRMHD conservation law the connection coefficient terms (source) terms occur. The error induced by these terms can be made arbitrarily small on a fine enough mesh compared to the differential terms. However, in practice the mesh might be coarse enough that the connection terms represent source terms that modify the solution of the conservation law in each time step (Del Zanna et al. 2007). This is not discussed further in this section which is concerned only with the Riemann solvers in GRMHD. The present paper does not focus on a consideration of stiff source terms.

**Figure 1:**
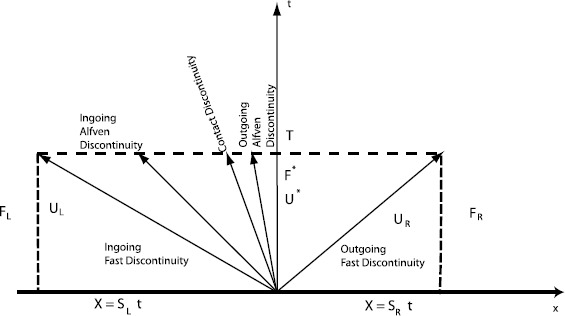
**Super Alfvenic Riemann fan.** An example of a one dimensional Riemann problem in which the flow is super-Alfvenic inward (to the left). The flow is not super-fast inward. This figure is used to illustrate what happens in a Riemann problem at the interface between two cells in a numerical scheme inside of the IACS. The 5-wave Riemann fan is illustrated (the slow waves are ignored without loss of generality) in order to show the difference of the resolved flux at the interface calculated with a 2-wave HLL Riemann solver and the same calculation performed with a 5-wave HLLI Riemann solver. Outgoing and ingoing are defined in the frame of reference of the plasma.

Consider the nature of the flow at the IACS. We are especially interested in the propagation of different RMHD wave families relative to the IACS which, in principle, could be stationary relative to the computational mesh. The space-time diagram is indicated in Figure 1. The flow is super-Alfvenic inward (to the left). The flow is not super-fast inward. This figure is a spacetime diagram of the MHD characteristics at an interface between cells inside of the IACS. The 5-wave Riemann fan is illustrated (the slow waves are ignored without loss of generality) in order to show the difference in the resolved flux that is produced by an HLL Riemann solver and a 5-wave based Riemann solver. The spacetime in Figure 1 is split into various zones. The world lines of isolated discontinuities that emanate from the zone boundary are shown in Figure 1. The resolved flux is the numerical flux in the zone that straddles the time axis. Inside of the IACS, the resolved flux is the numerical flux in the zone bounded on the left by Alfven wave that is outgoing in the proper frame (but ingoing on the computational mesh) and on the right by the outgoing fast wave. Physically, since the outgoing Alfven wave overlies the time axis in Figure 1, we are interested in capturing the stationary Alfvenic surface with maximum precision and the least possible numerical diffusion.

Consider a one-dimensional Riemann problem with left and right states $\mathbf{U}_{L}$ and $\mathbf{U}_{R}$ that are separated by a Riemann fan with extremal speeds that span $[S_{L}, S_{R}]$. Let the fluxes that correspond to the left and right states be given by $\mathbf{F}_{L}$ and $\mathbf{F}_{R}$. The numerical flux from practically any one-dimensional Riemann solver can be formally written as

2 $$ \mathbf{F}^{*} = \frac{1}{2}(\mathbf{F}_{L} + \mathbf{F}_{R}) -\frac{1}{2}\boldsymbol{\Theta}( \mathbf{U}_{R} - \mathbf{U}_{L}) . $$

The first term, which is the average of the left and right fluxes in the above expression, simply provides a centered, non-dissipative flux. The second term in the above expression is known as the dissipation term. The matrix, **Θ**, in the second term is the viscosity matrix, it regulates the dissipation of the Riemann solver. Further details on the ensuing mathematics can be found in Dumbser and Balsara (2016), Appendix B. Here we provide just enough results to show the difference between the dissipation from the HLL Riemann solver and the HLLI Riemann solver.

The viscosity matrix is usually expressed in terms of the right and left eigenvectors of the Roe matrix. Denoting the Roe matrix by $\mathbf{A}(\mathbf{U}_{L}, \mathbf{U}_{R})$, we will refer to its left and right eigenvectors by **L** and **R**. The eigenvalues of the Roe matrix will be denoted by a diagonal matrix, **Λ**. The viscosity matrix for the HLLI Riemann solver can be written as

3 $$ \boldsymbol{\Theta} =\mathbf{R}\boldsymbol{\Sigma}\mathbf{L} $$

with

4 $$ \boldsymbol{\Sigma} =\frac{S_{R} +S_{L}}{S_{R}-S_{L}}\boldsymbol{\Lambda} -2 \frac{S_{R}S_{L}}{S_{R}-S_{L}}\mathbf{I} +2\frac{S_{R}S_{L}}{S_{R}-S_{L}}\boldsymbol{\delta} . $$

Here **I** is the identity matrix and (for our purposes) ***δ*** is a special diagonal matrix that is introduced into the HLLI Riemann solver to judiciously reduce dissipation. Notice, therefore, that **Σ** is also a diagonal matrix. Please observe from the previous equation that the viscosity matrix introduces dissipation on a wave-by-wave basis, i.e., if the diagonal term corresponding to a particular wave becomes zero, the dissipation that is provided to that wave will also become zero. If we choose the diagonal terms in ***δ*** just right, we can minimize the dissipation and even guarantee that the dissipation of standing Alfven waves is exactly zero. This is exactly what has been done in Dumbser and Balsara (2016). Those authors provide precise expressions for the diagonal matrix, ***δ***, which ensure that stationary waves (whether they are Alfven waves or the entropy wave) have zero dissipation. For the sake of completeness, we catalogue their specification of the *i*th term of the diagonal matrix, ***δ***, as

5 $$ \delta_{i} = 1- \frac{\min(\lambda_{i}, 0)}{S_{L}}-\frac{\max(\lambda_{i}, 0)}{S_{R}} . $$

Here $\lambda_{i}$ is the *i*th eigenvalue of the Roe matrix corresponding to the wave that we are interested in. We see that the dissipation is finely tuned so that a moving Alfven wave gets just the minimum amount of dissipation that it needs, consistent with numerical stability. For example, a wave that propagates slowly relative to the computational mesh is given smaller dissipation compared to a wave that is propagating at high speed on the mesh. This decision to regulate the dissipation according to the wave speed is also what is demanded by numerical stability.

The viscosity matrix for the HLL Riemann solver is retrieved by setting $\boldsymbol{\delta} =0$. In that case, a standing Alfven wave has non-zero dissipation which means that the Alfven waves at the IACS surface will dissipate. Consequently, a numerical code that is based on the HLL Riemann solver (especially if it is operated at low to modest resolution) will not treat the IACS as a one-way surface with respect to the propagation of Alfven waves. We feel that this is a very important observation. Furthermore, with $\boldsymbol{\delta} =0$, it is easy to see that the HLL Riemann solver gives all waves a non-zero dissipation regardless of their wave speed. To see this, let $\lambda_{i}$ be a specific eigenvalue. Then the *i*th term for the diagonal matrix, **Σ** can be written as

6 $$ \varSigma _{i} = \frac{S_{R}(\lambda_{i}-S_{L})- S_{L}(S_{R}-\lambda_{i})}{S_{R}-S_{L}} . $$

For the sub-sonic case shown in Figure 1 we have $S_{L} < 0 < S_{R}$. We see that $\varSigma _{i} > 0$ for all intermediate eigenvalues, $\lambda_{i}$, with $S_{L} < \lambda_{i} < S_{R}$. Consequently all intermediate waves, like Alfven waves or contact discontinuities, will always be dissipated by the HLL Riemann solver.

In this section, we have only given a flavor of the dissipation characteristics of the HLLI Riemann solver and how it offers a substantial improvement over the HLL Riemann solver. The reader who is interested in details should please read Dumbser and Balsara (2016). The eigenvectors for RMHD that were used in this paper can all be obtained from Balsara (2001) or Antón et al. (2010).

## One dimensional Riemann solvers in RMHD

The Introduction has shown that it is very desirable to have Riemann solvers that can capture stationary, isolated contact discontinuities as well as stationary, isolated Alfven waves. Indeed the first of the one-dimensional Riemann solvers for RMHD by Komissarov (1999) and Balsara (2001) were Roe-type Riemann solvers. Because such Riemann solvers retain the entire set of eigenvectors for the RMHD system, they can indeed capture stationary, isolated contact discontinuities as well as stationary, isolated Alfven waves on a mesh. There has been a recent effort by Antón et al. (2010) to revive the use of Roe-type Riemann solvers in RMHD simulations, but the effort has met with limited success owing to the exorbitant computational cost of such Riemann solvers, especially for RMHD. The Roe-type Riemann solvers also have an inherent deficiency. This has to do with their loss of positivity of density and pressure in certain circumstances (Einfeldt 1988; Einfeldt et al. 1991). It is easy to find mentions in the early literature on RMHD (Komissarov 1999, 2004, 2005), showing that the early RMHD simulation codes struggled with positivity issues and, therefore, reverted to the use of the HLL Riemann solver. This issue is relevant to the extremely low density environment of event horizon magnetospheres.

HLLD Riemann solvers. Mignone et al. (2009), also enable a code to capture stationary, isolated contact discontinuities as well as stationary, isolated Alfven waves. But they have their own set of attendant problems, as discussed in the Introduction. HLLC Riemann solvers (Mignone and Bodo 2006; Honkkila and Janhunen 2007; Kim and Balsara 2014), represent a compromise position where they enable a code to capture stationary, isolated contact discontinuities, but not Alfven waves. The HLLI Riemann solver of Dumbser and Balsara (2016) is built on top of an HLL Riemann solver, so it inherits all the beneficial positivity properties of the HLL Riemann solver. However, it introduces sub-structure in the Riemann fan. Typically, that substructure includes the contribution from eigenvectors of the contact discontinuity and eigenvectors associated with Alfven waves. The eigenvectors for the fast and slow magnetosonic waves are very expensive to evaluate computationally, and their evaluation is avoided in the HLLI Riemann solver. As a result, the HLLI Riemann solver enables a code to capture stationary, isolated contact discontinuities as well as stationary, isolated Alfven waves at a very low computational cost. Unlike HLLC and HLLD, the HLLI Riemann solver does not require an iterative solution, thereby ensuring that it has even lower computational cost. In the next few paragraphs we demonstrate this facet of the HLLI Riemann solver for RMHD.

**Figure 2:**
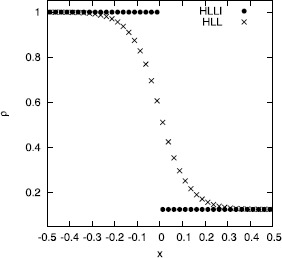
**HLL Riemann solver vs. HLLI Riemann solver stationary Alfven discontinuity.** Figure 2 shows two simulations of an isolated, stationary contact discontinuity. The density variable is shown. The result from the HLL Riemann solver is shown with crosses while the result from the HLLI Riemann solver is shown with dots.

Figure 2 shows two simulations of an isolated, stationary contact discontinuity as suggested by Honkkila and Janhunen (2007). The density variable is shown. We use the same parameters as the previous authors and we run the simulation to a final time that is ten times larger than the one suggested by Honkkila and Janhunen. The result from the HLLI Riemann solver is shown with filled dots, the result from the HLL Riemann solver is shown with crosses. We see that the HLL Riemann solver has produced significant dissipation of the contact discontinuity, while the HLLI Riemann solver has captured the contact discontinuity exactly.

**Figure 3:**
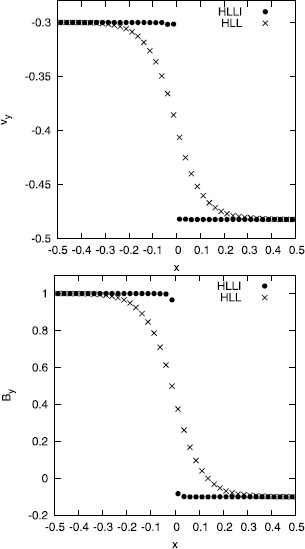
**HLL Riemann solver vs. HLLI Riemann solver contact discontinuity.** Figure 3 shows two simulations of an isolated, stationary Alfven wave. The transverse velocity and magnetic field are shown. The result from the HLL Riemann solver is shown with crosses while the result from the HLLI Riemann solver is shown with dots.

Figure 3 shows two simulations of an isolated, stationary Alfven wave. The transverse velocity and magnetic field are shown. This problem was suggested by Mignone et al. (2009) and we use the same parameters as the previous authors but we run the simulation to a final time that is four times larger than the final time quoted by Mignone et al. (2009). The result from the HLLI Riemann solver is shown with filled dots, the result from the HLL Riemann solver is shown with crosses. As before, we see that the HLL Riemann solver has produced significant dissipation of the Alfven wave discontinuity, while the HLLI Riemann solver has captured the Alfven wave discontinuity exactly. Figures 2 and 3 both used a standard, second order scheme, the only difference being the use of the HLLI Riemann solver.

## Multidimensional propagation of Alfven waves

Figures 2 and 3 showed that the one-dimensional HLLI Riemann solver can dramatically reduce the dissipation compared to the HLL Riemann solver. Multidimensional treatment of Alfven waves on a computational mesh requires a multidimensional Riemann solver. In Balsara (2004), we were able to formulate a test problem that measures the ability of a multidimensional MHD code to propagate Alfven waves with the least amount of dissipation. It has been shown that this test problem is very important in benchmarking the dissipation characteristics of multidimensional codes for classical MHD. The analogous problem which benchmarks the low dissipation propagation of Alfven waves in RMHD has been recently presented in Balsara and Kim (2016). It consists of torsional Alfven waves propagating obliquely to the mesh lines of a two-dimensional mesh. The mesh has $120\times 120$ zones. We do not repeat the problem description. Instead, we show the results and intercompare with older methods that involve dissipation doubling from Gardiner and Stone (2005, 2008).

**Figure 4:**
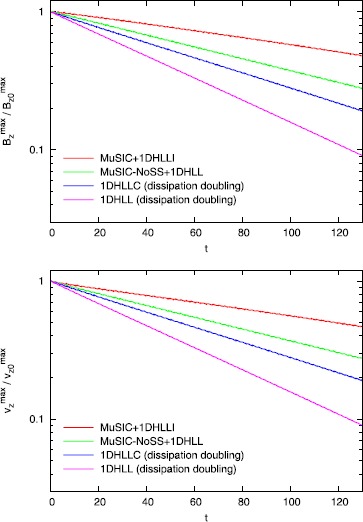
**Torsional Alfven wave dissipation test.** Figure 4 shows the results of the torsional Alfven wave dissipation test. A second order WENO reconstruction was used in all these tests. Figure 4a shows the decay in the *z*-component of the velocity as a function of time. Figure 4b shows the same for the *z*-component of the magnetic field.

Figure 4 shows the results of the torsional Alfven wave dissipation test from Balsara and Kim (2016). In Figure 4, we use the same second order reconstruction algorithm from the RMHD version of the RIEMANN code. Figure 4a shows the decay in the *z*-component of the velocity of the Alfven wave as a function of time. Figure 4b shows the same for the *z*-component of the magnetic field of the Alfven wave. The vertical axis is logarithmically scaled. A faster rate of decline in Figure 4 indicates that the associated numerical scheme has higher dissipation. The curve that is labeled ‘$\mathrm{MuSIC}+1\mathrm{DHLLI}$’ uses the one-dimensional HLLI Riemann solver at the zone faces and the MuSIC Riemann solver with sub-structure at the zone edges. We see that it displays minimal dissipation. This is because the MuSIC Riemann solver is designed to be the exact, multidimensional analogue of the one-dimensional HLLI Riemann solver. The curve that is labeled ‘$\mathrm{MuSIC}\mbox{-}\mathrm{NoSS}+1\mathrm{DHLLI}$’ uses the same algorithmic combination with one simple exception. The MuSIC Riemann solver is prevented from endowing sub-structure to the strongly-interacting state. We see that when the sub-structure in the MuSIC Riemann solver is artificially removed, the dissipation of Alfven waves increases. This makes the very nice point that all facets of the newly designed MuSIC Riemann solver play a role in reducing dissipation. It is very useful to cross-compare with the dissipation doubling ideas from Gardiner and Stone (2005, 2008). The curve that is labeled ‘1D HLLC (dissipation doubling)’ doubles the dissipation in the HLLC Riemann solver using the ideas from Gardiner and Stone (2005, 2008). Despite the one-dimensional HLLC Riemann solver being an able performer, we see that it dramatically increases the dissipation that is provided to the torsional Alfven waves. Lastly, one is most interested in understanding what happens when the dissipation doubling ideas from Gardiner and Stone (2005, 2008) are applied to the one-dimensional HLL Riemann solver. This is shown in Figure 4 by the curve labeled ‘1D HLL (dissipation doubling)’. We see that Alfven waves are strongly dissipated.

**Figure 5:**
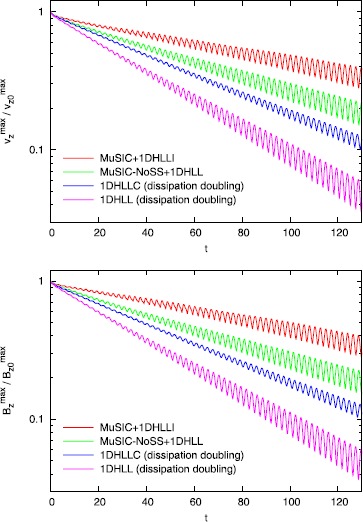
**MC limiter.** Figure 5 is analogous to Figure 4 with the exception than an MC limiter was used. The MC limiter is considered inferior to a good WENO scheme. Comparing Figures 4 and 5, this observation is apparent in the figures.

It should also be emphasized that the reconstruction that was used in Figure 4 is the linear part of the $r=3$ WENO reconstruction. This is already a very superior reconstruction strategy. It is almost as superior as a true third order reconstruction strategy. It is quite possible that reconstruction is done with a second order TVD limiter, like the MC limiter. In that case, the analogous results are shown in Figure 5. We see considerably increased dissipation in Figure 5 compared to Figure 4.

**Figure 6:**
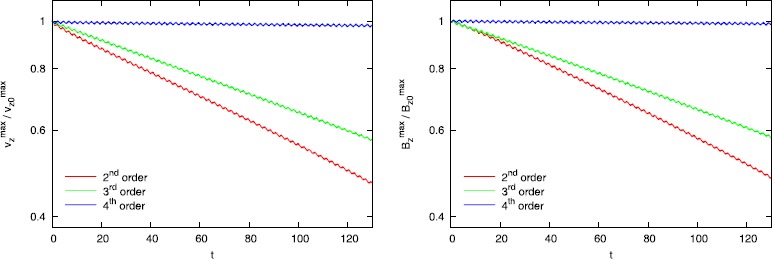
**ADER-WENO Schemes the 2D MuSIC Riemann solver.** Figure 6 shows the same Alfven wave propagation test. This time, we used different ADER-WENO schemes with increasing order of accuracy. We also used the 1D HLLI Riemann solver along with the 2D MuSIC Riemann solver with sub-structure. We see that higher order schemes produce lower dissipation.

In Balsara and Kim (2016), ADER-WENO schemes were designed that go all the way up to fourth order of accuracy. It is, therefore, very interesting to ask whether improved accuracy gives us an improved result for the propagation of torsional Alfven waves. Figure 6 shows the propagation of torsional Alfven waves when second, third and fourth order ADER-WENO schemes are used. All these schemes used the one-dimensional HLLI Riemann solver at the zone faces and the MuSIC Riemann solver with sub-structure at the zone edges. We clearly see that the higher order schemes show vastly reduced dissipation. In Balsara and Kim (2016), we also demonstrate that modern high order schemes perform robustly even in the vicinity of strong shocks. Thus the barrier to their use in astrophysics is dramatically reduced by this work.

**Figure 7:**
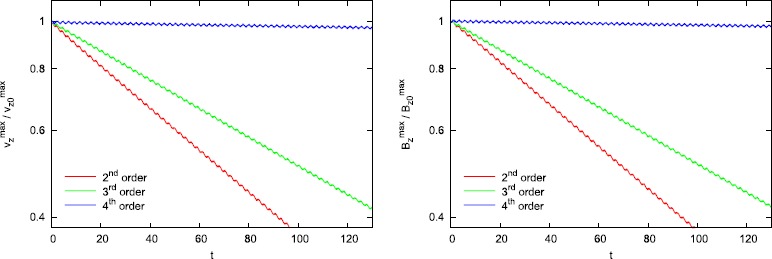
**ADER-WENO schemes and the 2D HLL Riemann solver.** Figure 7 shows the same Alfven wave propagation test as Figure 6 but when a lower quality Riemann solver is used. We used the same ADER-WENO schemes as in Figure 6. The only difference is that the simulations in this Figure were run with a 1D HLL Riemann solver and a 2D HLL Riemann solver. Comparing Figure 6 to Figure 7 enables us to appreciate the improvement that Riemann solvers with sub-structure provide in reducing dissipation.

Figure 6 clearly shows us that the combination of a high accuracy method and 1D and 2D Riemann solvers that preserve sub-structure produces very low dissipation. This is especially apparent in the fourth order simulation shown in Figure 6. Out of curiosity, we can always ask what fraction of the improvement in Alfven wave propagation stems from the use of a higher order scheme and what fraction of the improvement in Alfven wave propagation stems from the use of Riemann solvers that retain substructure? For that reason, the same simulations from Figure 6 were run again with 1D and 2D Riemann solvers that do not preserve sub-structure. The results are shown in Figure 7. In other words, for Figure 7, the 1D Riemann solver was an HLL Riemann solver and the 2D Riemann solver was a 2D analogue of an HLL Riemann solver. Consequently, Figure 7 shows the result of Alfven wave propagation when lower quality Riemann solvers are used. We see that the second order result in Figure 7 is substantially more dissipative than the second order result from Figure 6. We also see that the second order result from Figure 6 has a dissipation that is comparable to the third order result from Figure 7. In other words, using a Riemann solver with sub-structure produces a very palpable improvement in second and third order schemes. When we compare the fourth order results from Figures 6 and 7, we see that they are indeed quite comparable. In other words, we suggest that at fourth and higher orders of accuracy the value of a Riemann solver that preserves sub-structure is diminished because the fourth order reconstruction itself is so very accurate! Note though that a third order scheme will typically be two to three times more expensive compared to a second order scheme. Similarly, a fourth order accurate scheme will be about three times more expensive compared to a third order scheme. For that reason, it is very profitable to try and extract as much performance and quality from a lower order scheme, especially if one does not have access to a higher order scheme.

Figures 6 and 7 show that there is always a small wiggle in the maximum amplitude of the Alfven waves. This wiggle stems from the fact that the Alfven waves in our test problem have large amplitude and are, therefore, prone to small initialization errors or errors in start-up transients that never go away. The non-relativistic analogue of this test problem also shows the same issue. The wiggles make the test problem used in Figure 6 an inadequate test problem for demonstrating order of accuracy. That is especially true at fourth order where the wiggles have an amplitude that is comparable to the decay of the Alfven wave. (In Balsara and Kim (2016) we do present better test problems for demonstrating the accuracy of an RMHD scheme.) However, please observe from Figure 6 that at second and third orders of accuracy, the rate of decay in the Alfven wave amplitude is much larger than the wiggles, so that it can be used to document the second and third orders of accuracy of the schemes used here. Table 1 shows the $L_{1}$ error in the *z*-momentum density and the *z*-component of the magnetic field as a function of mesh resolution at the second order. Table 2 shows the $L_{1}$ error in the *z*-momentum density and the *z*-component of the magnetic field as a function of mesh resolution at the third order. The errors are shown at a time of 30 units, by which point the decay in the Alfven waves at second and third orders of accuracy is much larger than the amplitude of the wiggles. We see that the second and third order accurate methods do indeed achieve their design accuracies.

**Table 1:** $\pmb{L_{1}}$ **error in the** ***z*** **-momentum density and the** ***z*** **-component of the magnetic field as a function of mesh resolution at the second order**

**Zones**	$\boldsymbol{L_{1}}$ **error** ***z*** **-momentum density**	**Accuracy**	$\boldsymbol{L_{1}}$ **error** ***z*** **-magnetic field**	**Accuracy**
60 × 60	2.7693 × 10^−1^		1.1971 × 10^−1^	
120 × 120	7.5660 × 10^−2^	1.87	3.3246 × 10^−2^	1.85
240 × 240	1.6083 × 10^−2^	2.23	7.1989 × 10^−3^	2.21

**Table 2:** $\pmb{L_{1}}$ **error in the** ***z*** **-momentum density and the** ***z*** **-component of the magnetic field as a function of mesh resolution at the third order**

**Zones**	$\boldsymbol{L_{1}}$ **error** ***z*** **-momentum density**	**Accuracy**	$\boldsymbol{L_{1}}$ **error** ***z*** **-magnetic field**	**Accuracy**
60 × 60	2.1295 × 10^−1^		9.2981 × 10^−2^	
120 × 120	3.7593 × 10^−2^	2.50	1.6720 × 10^−2^	2.48
240 × 240	5.1524 × 10^−3^	2.87	2.2025 × 10^−3^	2.92

## Discussion and future prospects

In this article, we describe subtle points uniquely associated with numerical simulations of event horizon magnetospheres. All inflows must pass through an IACS, thereby rendering the interior region out of Alfven wave communication with the out-flowing wind. Thus, the IACS is a charge horizon (a one-way membrane) for the global flow since the Alfven wave is the only wave that carries a physical charge (Punsly 2004). The IACS causally excises most of the active region of space-time (the ergosphere) that would be the putative element to enforce rapid rotation of the system. The implication is that simulations of event horizon magnetospheres should be performed with numerical schemes that represent the Alfvenic properties of the system with high fidelity.

It was demonstrated that an improvement of numerical accuracy can be attained by utilizing numerical schemes based on HLLI and MuSIC Riemann solvers rather than schemes based on 1-D HLL solvers. In particular, for 1-D RMHD solvers, the HLLI Riemann solver provides the theoretical minimum dissipation of the Alfven wave and contact discontinuities that can still ensure a numerically stable scheme. In Section 4, we discussed the multidimensional extension of HLLI RMHD Riemann solver; the RMHD MuSIC Riemann solver. It is in higher dimensions that we see an even larger improvement over schemes based on 1-D HLL solvers. The MuSIC Riemann solver was shown to significantly reduce the dissipation of Alfven waves in large part to its ability to resolve the strongly interacting region that is typically ignored in schemes based on 1-D HLL Riemann solvers. We also show that very high order schemes might be free of the excessive dissipation that arises from a lower quality Riemann solver. However, that transition occurs only when schemes of fourth and higher order of accuracy are used.

Low Alfven and contact discontinuity dissipation in a numerical scheme, such as those based on MuSIC, should allow the proper propagation of Alfven wave information in the following unique circumstances endemic to event horizon magnetospheres that were discussed in the Introduction.

1. At the risk of being repetitive, the paired wind systems evolves outward and inward towards two asymptotic infinities as opposed to having a causal MHD boundary at one terminus. Thus, unlike other MHD wind problems there is a more complex critical point structure. Most specifically, all inflows pass through the inner Alfven critical surface. Thereby causally disconnecting the outflowing wind from Alfven radiation emanating from the majority of the rapidly rotating ergospheric plasma. The Alfven wave is the only wave that propagates a physical charge and thus should be involved in the establishment of the Goldreich-Julian charge density or equivalently, the field line rotation rate, $\varOmega _{F}$. Thus, reducing the numerical dissipation of the Alfven wave by implementing the HLLI or MuSIC Riemann solvers would seem to help in this regard.
2. The paired wind system constantly drains itself of plasma in the MHD limit. Thus, plasma injection by means of a mass floor is required. This process will dissipate MHD waves generated by the MHD system and inject new MHD waves. The process is nontrivial and has been shown to modify $\varOmega _{F}$ significantly in certain 3-D simulations. Since, in principle, it can modify $\varOmega _{F}$ and mass injection perturbs the Alfven waves generated in the system, a Riemann solver lowers Alfven dissipation might shed light on the nature of the transients that occur as different plasma injection scenarios are explored.
3. Another related issue is the large numerical diffusion of plasma from the bounding accretion disk into the event horizon magnetosphere. Diffusion is substantially reduced if the Riemann solver respects the contact discontinuity. Since the source of plasma injection might be important to the establishment of $\varOmega _{F}$ this is an important issue as well. Riemann solvers, such as the HLLI or MUSIC Riemann solvers, which treat contact discontinuities explicitly can help in this regard.

In this article, we presented both theoretical and numerical arguments that support the notion that numerical schemes based on the multi-dimensional MuSIC Riemann solver can potentially provide an improvement over existing methods of modeling 3-D event horizon magnetospheres. In particular, the MuSIC Riemann solver is both well suited for the low density environment endemic to the event horizon magnetosphere and it provides reduced dissipation of the Alfven and contact discontinuities. It is also computationally efficient which offsets the cost of improving the computational accuracy.

In Balsara and Kim (2016) we provide a subluminal scheme for RMHD as well as a discussion of the MuSIC Riemann solver. The reader who wishes to get further information can also visit the second author’s website http://www.nd.edu/~dbalsara/Numerical-PDE-Course.

## References

[CR1] Antón L, Miralles JA, Martí JM (2010). Relativistic magnetohydrodynamics: renormalized eigenvectors and full wave decomposition Riemann solver. Astrophys. J. Suppl. Ser..

[CR2] Balsara D (2001). Total variation diminishing scheme for relativistic magnetohydrodynamics. Astrophys. J. Suppl. Ser..

[CR3] Balsara DS (2004). Schemes for magnetohydrodynamics with divergence-free reconstruction. Astrophys. J. Suppl. Ser..

[CR4] Balsara DS (2010). Multidimensional extension of the HLLE Riemann solver; application to Euler and magnetohydrodynamical flows. J. Comput. Phys..

[CR5] Balsara DS (2012). A two-dimensional HLLC Riemann solver for conservation laws: application to Euler and magnetohydrodynamic flows. J. Comput. Phys..

[CR6] Balsara DS (2014). Multidimensional Riemann problem with self-similar internal structure. Part I - application to hyperbolic conservation laws on structured meshes. J. Comput. Phys..

[CR7] Balsara DS (2015). Three dimensional HLL Riemann solver for conservation laws on structured meshes; application to Euler and magnetohydrodynamic flows. J. Comput. Phys..

[CR8] Balsara DS, Dumbser M (2015). Multidimensional Riemann problem with self-similar internal structure. Part II - application to hyperbolic conservation laws on unstructured meshes. J. Comput. Phys..

[CR9] Balsara DS, Dumbser M, Abgrall R (2014). Multidimensional HLL and HLLC Riemann solvers for unstructured meshes - with application to Euler and MHD flows. J. Comput. Phys..

[CR10] Balsara DS, Kim J (2016). A subluminal relativistic magnetohydrodynamics scheme with ADER-WENO predictor and multidimensional Riemann solver-based corrector. J. Comput. Phys..

[CR11] Balsara, DS, Nkonga, B, Dumbser, M, Munz, CD: (2016b, in preparation)

[CR12] Balsara DS, Vides J, Gurski K (2016). A two-dimensional Riemann solver with self-similar sub-structure - alternative formulation based on least squares projection. J. Comput. Phys..

[CR13] Beckwith K, Stone JM (2011). A second-order Godunov method for multi-dimensional relativistic magnetohydrodynamics. Astrophys. J. Suppl. Ser..

[CR14] Beskin VS, Zheltoukhov AA (2103). On the structure of the magnetic field near a black hole in active galactic nuclei. Astron. Lett..

[CR15] De Villiers J-P, Hawley JF, Krolik JH (2003). Magnetically driven accretion flows in the Kerr metric. I. Models and overall structure. Astrophys. J..

[CR16] Del Zanna L, Zanotti O, Bucciantini N, Londrillo P (2007). ECHO: a Eulerian conservative high-order scheme for general relativistic magnetohydrodynamics and magnetodynamics. Astron. Astrophys..

[CR17] Dumbser M, Balsara D (2016). A new efficient formulation of the HLLEM Riemann solver for general conservative and non-conservative hyperbolic systems. J. Comput. Phys..

[CR18] Einfeldt B (1988). On Godunov-type methods for gas dynamics. SIAM J. Numer. Anal..

[CR19] Einfeldt B, Munz C-D, Roe P, Sjogreen B (1991). On Godunov-type methods near low densities. J. Comput. Phys..

[CR20] Etienne Z, Paschalidis V, Haas R, Mösta P, Shapiro S (2015). IllinoisGRMHD: an open-source, user-friendly GRMHD code for dynamical spacetimes. Class. Quantum Gravity.

[CR21] Fragile PC, Blaes OM, Anninos P, Salmonson JD (2007). Global general relativistic magnetohydrodynamic simulation of a tilted black hole accretion disk. Astrophys. J..

[CR22] Gammie CF, McKinney JC, Toth G (2003). A numerical scheme for general relativistic magnetohydrodynamics. Astrophys. J..

[CR23] Gardiner TA, Stone JM (2005). An unsplit Godunov method for ideal MHD via constrained transport. J. Comput. Phys..

[CR24] Gardiner TA, Stone JM (2008). An unsplit Godunov method for ideal MHD via constrained transport in three dimensions. J. Comput. Phys..

[CR25] Giacomazzo B, Rezzolla L (2006). The exact solution of the Riemann problem in relativistic magnetohydrodynamics. J. Fluid Mech..

[CR26] Giacomazzo B, Rezzolla L (2007). WhiskyMHD: a new numerical code for general relativistic magnetohydrodynamics. Class. Quantum Gravity.

[CR27] Hawley J, Krolik K (2006). Magnetically driven jets in the Kerr metric. Astrophys. J..

[CR28] Honkkila V, Janhunen P (2007). HLLC solver for ideal relativistic MHD. J. Comput. Phys..

[CR29] Kappeli R, Mishra S (2014). Well-balanced schemes for the Euler equations with gravitation. J. Comput. Phys..

[CR30] Kappeli R, Mishra S (2016). A well-balanced finite volume scheme for the Euler equations with gravitation. The exact preservation of hydrostatic equilibrium with arbitrary entropy stratification. Astron. Astrophys..

[CR31] Kim J, Balsara DS (2014). A stable HLLC Riemann solver for relativistic magnetohydrodynamics. J. Comput. Phys..

[CR32] Komissarov S (1999). A Godunov-type scheme for relativistic magnetohydrodynamics. Mon. Not. R. Astron. Soc..

[CR33] Komissarov S (2004). General relativistic magnetohydrodynamic simulations of monopole magnetospheres of black holes. Mon. Not. R. Astron. Soc..

[CR34] Komissarov S (2005). Observations of the Blandford-Znajek process and the magnetohydrodynamic Penrose process in computer simulations of black hole magnetospheres. Mon. Not. R. Astron. Soc..

[CR35] Krolik K, Hawley J, Hirose S (2005). Magnetically driven accretion flows in the Kerr metric. IV. Dynamical properties of the inner disk. Astrophys. J..

[CR36] McKinney J, Blandford R (2009). Stability of relativistic jets from rotating, accreting black holes via fully three-dimensional magnetohydrodynamic simulations. Mon. Not. R. Astron. Soc. Lett..

[CR37] McKinney J, Tchekhovskoy A, Blandford R (2012). General relativistic magnetohydrodynamic simulations of magnetically choked accretion flows around black holes. Mon. Not. R. Astron. Soc..

[CR38] Mignone A, Bodo G (2006). An HLLC Riemann solver for relativistic flows - II. Magnetohydrodynamics. Mon. Not. R. Astron. Soc..

[CR39] Mignone A, Ugliano M, Bodo G (2009). A five-wave Harten-Lax-van Leer Riemann solver for relativistic magnetohydrodynamics. Mon. Not. R. Astron. Soc..

[CR40] Parés C (2006). Numerical methods for nonconservative hyperbolic systems: a theoretical framework. SIAM J. Numer. Anal..

[CR41] Punsly B (2004). Fast-wave polarization charge horizons, and the time evolution of force-free magnetospheres. Astrophys. J..

[CR42] Punsly B (2008). Black Hole Gravitohydromagnetics.

[CR43] White, CJ, Stone, JM: GRMHD in Athena++ using advanced Riemann solvers and staggered-mesh constrained transport (2015). arXiv:1511.00943

[CR44] Zanotti O, Dumbser M (2016). Efficient conservative ADER schemes based on WENO reconstruction and space-time predictor in primitive variables. Comput. Astrophys. Cosmol..

